# CircWAC induces chemotherapeutic resistance in triple-negative breast cancer by targeting miR-142, upregulating WWP1 and activating the PI3K/AKT pathway

**DOI:** 10.1186/s12943-021-01332-8

**Published:** 2021-03-01

**Authors:** Lei Wang, Yehui Zhou, Liang Jiang, Linlin Lu, Tiantian Dai, Aoshuang Li, Yan Chen, Lifeng Zhang

**Affiliations:** 1 Department of General Surgery, The First Affiliated Hospital of Soochow University, 188 Shizi Street, Suzhou, 215000 China; 2 Insitute of Blood Transfusion, Suzhou Blood Center, Suzhou, 215000 China

**Keywords:** circWAC, miR-142, WWP1, PI3K/AKT, TNBC

## Abstract

**Background:**

Chemotherapeutic resistance is the main cause of clinical treatment failure and poor prognosis in triple-negative breast cancer (TNBC). There is no research on chemotherapeutic resistance in TNBC from the perspective of circular RNAs (circRNAs).

**Methods:**

TNBC-related circRNAs were identified based on the GSE101124 dataset. Quantitative reverse transcription PCR was used to detect the expression level of circWAC in TNBC cells and tissues. Then, in vitro and in vivo functional experiments were performed to evaluate the effects of circWAC in TNBC.

**Results:**

CircWAC was highly expressed in TNBC and was associated with worse TNBC patient prognosis. Subsequently, it was verified that downregulation of circWAC can increase the sensitivity of TNBC cells to paclitaxel (PTX) in vitro and in vivo. The expression of miR-142 was negatively correlated with circWAC in TNBC. The interaction between circWAC and miR-142 in TNBC cells was confirmed by RNA immunoprecipitation assays, luciferase reporter assays, pulldown assays, and fluorescence in situ hybridization. Mechanistically, circWAC acted as a miR-142 sponge to relieve the repressive effect of miR-142 on its target WWP1. In addition, the overall survival of TNBC patients with high expression of miR-142 was significantly better than that of patients with low expression of miR-142, and these results were verified in public databases. MiR-142 regulated the expression of WWP1 and the activity of the PI3K/AKT pathway. It was confirmed that WWP1 is highly expressed in TNBC and that the prognosis of patients with high WWP1 expression is poor.

**Conclusions:**

CircWAC/miR-142/WWP1 form a competing endogenous RNA (ceRNA) network to regulate PI3K/AKT signaling activity in TNBC cells and affect the chemosensitivity of cells.

**Supplementary Information:**

The online version contains supplementary material available at 10.1186/s12943-021-01332-8.

## Background

As a malignancy arising in breast epithelial tissue, approximately 99% of breast cancers occurs in women. According to GLOBOCAN data, in 2018, there were approximately 2.1 million new cases of breast cancer worldwide, accounting for 11.6% of the total number of cancer diagnoses and approximately 630,000 deaths, accounting for 6.6% of the total number of cancer deaths; thus, breast cancer had the highest incidence among female cancers [1]. The incidence and mortality rates of breast cancer in China increased annually from 2003 to 2014, seriously affecting women’s health and life expectancy [2, 3]. In China, there were approximately 270,000 new cases of breast cancer and 70,000 deaths in 2015 [4]. Triple-negative breast cancer (TNBC), accounting for 15–20% of breast cancers, is one of the most unique types of breast cancer, and its biological behavior, onset characteristics and prognosis are different from those of other molecular subtypes of breast cancer [5]. TNBC tissues are negative for ER, PR and HER-2; thus, their sensitivity to endocrine therapies, molecular targeted therapies and chemotherapeutics is reduced, increasing the difficulty of clinical treatment. To date, surgery, radiotherapy and chemotherapy are the main treatments for TNBC. In clinical practice, adjuvant chemotherapy, such as anthracycline and paclitaxel (PTX)-based chemotherapy regimens, is still the main treatment method, but the development of chemotherapeutic resistance is still the main cause of clinical treatment failure and poor prognosis in breast cancer patients, and the 5-year survival rate of TNBC is significantly lower than that of non-TNBC breast cancers [6]. Therefore, it is extremely urgent to improve the efficacy of TNBC therapies and prolong the patient survival time.

Currently, with the development of high-throughput sequencing techniques, RNomics has gradually become a focus of attention. Circular RNAs (circRNAs) are a new type of RNA molecule that differ from traditional linear RNAs. They have a closed loop structure and are abundant in eukaryotic transcriptomes. CircRNAs are usually formed by cleavage and circularization of host gene exons or introns. Their closed loop structure is not sensitive to RNase R; thus, they are more stable than linear RNAs and can be used as tumor markers and potential targets in clinical applications [7]. In recent studies, it has been found that circRNAs play an important regulatory role in tumorigenesis and tumor development. For example, ciRS-7 upregulates the expression of oncogenes such as mTOR, EGFR and PIK3CD by sponging miR-7 and participates in the progression of neuroblastoma and renal cancer [8]. Research on breast cancer-related circRNAs is in its infancy [9], and circRNAs related to chemosensitivity in TNBC have not been reported. Therefore, further exploration of chemotherapeutic resistance in TNBC from the perspective of circRNAs will help reveal novel mechanisms underlying the occurrence and development of PTX resistance in TNBC, and it can provide new ideas for clinical diagnosis and treatment.

In the present study, the differentially expressed circRNAs in TNBC were screened, and TNBC-related circRNAs were identified based on the GSE101124 dataset [10]. Combining these results with the results in TNBC patient specimens from the First Affiliated Hospital of Soochow University, it was found that circWAC was highly expressed in TNBC and that its expression level was positively correlated with the prognosis of TNBC; that is, the higher the expression of circWAC, the worse was the prognosis of TNBC patients. In vivo and in vitro experiments confirmed that downregulation of circWAC in TNBC increased the sensitivity of cells to PTX chemotherapy. Subsequently, mechanistically, it was confirmed that circWAC indirectly upregulated the expression of WWP1, the target gene of miR-142, and activated the PI3K/AKT pathway by competitively binding to miR-142, thus leading to chemotherapeutic resistance in TNBC.

## Methods

### TNBC tissue samples

The tissue samples used in this study were obtained from TNBC patients undergoing surgical treatment at the First Affiliated Hospital of Soochow University. The surgical specimens were cryopreserved in liquid nitrogen after isolation. Thirty paired TNBC tumor/paracancer tissue samples were randomly selected to detect the differential expression of circRNAs (none of the patients received any other treatments, including radiotherapy, chemotherapy or targeted drug therapy, prior to surgery). In addition, 90 TNBC patients who underwent radical mastectomy between 2013 and 2015 at the First Affiliated Hospital of Soochow University and received at least 4 cycles of PTX adjuvant chemotherapy were selected. Postoperative pathological diagnostic reporting was performed by two pathologists from the First Affiliated Hospital of Soochow University. The study patients and their families were informed of the research during the preoperative conversation, and a doctor-patient informed consent form was signed. The research project complied with the *Declaration of Helsinki* and was approved by the Ethics Committee of the First Affiliated Hospital of Soochow University.

### Cell lines

The cell lines MDA-MB-231, MDA-MB-468, HCC-1937, MDA-MB-361, MCF-7 and MCF-10A used in this experiment were purchased from ATCC. Cells were cultured in an incubator at 37 °C with 5% CO_2_ and saturated humidity.

### Quantitative reverse transcription-polymerase chain reaction (qRT-PCR)

TRIzol reagent was used to extract total RNA from tissues and cells, and a NanoDrop 2000 microspectrophotometer was used to determine the quality and concentration of total RNA. A PrimeScript RT Reagent Kit was used for reverse transcription to synthesize cDNA. Fluorescence quantitative PCR was performed using TB Green Fast qPCR Mix. All primers were designed and synthesized by Tingzhou Bio (Shanghai, China). Related primer sequences are shown in Supplementary Table 1.

### Western blot analysis

Total cellular protein was extracted, and protein quantification was performed with a protein quantification kit. After quantification, 20 μg of the samples was loaded on a 12% SDS-PAGE gel, separated, transferred to a PVDF membrane, blocked with skim milk (50 g/L), washed, and incubated with primary antibodies (1:500) and secondary antibodies (1:2000), and immunoreactions were then visualized with ECL reagents. With the GAPDH and β-actin proteins as internal references, the optical density (OD) values of relevant proteins (WWP1, PTEN, AKT, pS473-AKT, etc.) were analyzed using Quantity One software.

### Cell transfection

The circWAC expression plasmid, circWAC small interfering RNA (siRNA), WWP1 siRNA, control constructs, negative control miRNA (miR-NC), miR-142 mimic, and miR-142 inhibitor were purchased from XimaoBio (Shanghai, China). According to the instructions, cells were transfected using Lipofectamine 3000.

### Dual luciferase reporter assay

To experimentally predict the possible miRNA target genes and binding sequences, the wild-type and mutant sequences were synthesized and cloned into dual luciferase reporter plasmids (Promega) containing the psiCheck2 promoter. After MDA-MB-231 cells were inoculated into a 96-well plate and cultured for 24 h, they were cotransfected with the wild-type or mutant reporter gene plasmids and overexpression or silencing plasmid mimics. Luciferase activity was measured 48 h after transfection.

### CCK-8 assay

Cells in logarithmic growth phase were fully and evenly spread in a 96-well plate and cultured for 24 h. After 24 h of drug treatment and/or transfection, the medium was replaced, and CCK-8 reagent at a final concentration of 10% was then added to the cultures. After 1–4 h, the absorbance at 450 nm was measured using a microplate reader. Cell survival curves were plotted with the cell survival rate as the ordinate and the PTX concentration as the abscissa.

### Colony formation assay

Cells were inoculated into a 6-well plate, the medium was replaced after 24 h of drug treatment and/or transfection, and the cells were then fixed with methanol for 15 min. Then, the fixative was removed, and Giemsa staining was performed for 20 min.

### Apoptosis assay

A FITC Annexin V Apoptosis Assay Kit was used for apoptosis detection as follows. Cells in logarithmic growth phase were collected, resuspended and evenly spread in a 6-well plate, and cultured for 24 h. After 24 h of transfection and/or drug treatment, the supernatant was removed, and the cells were then washed twice with PBS, trypsinized and resuspended in HEPES buffer (containing calcium). Then, the tumor cells were stained with Annexin V-FITC and PI for 15 min in the dark. After washing, apoptosis was detected by flow cytometry.

### Cell invasion and metastasis assays

After 24 h of transfection treatment, the cells were prepared into a cell suspension (1 × 10^5^/mL) with culture medium. One hundred microliters of the suspension was collected and inoculated into the upper Transwell chamber, and 500 μL of medium containing 10% fetal bovine serum was added into the lower Transwell chamber. After 24 h of incubation at 37 °C, the cells in the upper chamber were wiped off with cotton swabs; the cells in the lower chamber were fixed with 4% paraformaldehyde, and their nuclei were stained with DAPI. Five fields were randomly selected under a microscope (100× magnification) to calculate the average number of migrated cells. In the cell invasion assay, the membrane in the upper Transwell chamber was coated with prediluted Matrigel, and the plate was incubated at room temperature for 6 h. The remaining steps were the same as those in the cell migration assay.

### Fluorescence in situ hybridization (FISH)

FISH was performed on MDA-MB-231 cells to determine the subcellular location of circWAC and miR-142. The cell specimens were placed in PBS containing 10% fixative solution for 5 min for predenaturation. The slide specimens were immersed in the fixative solution twice for 10 min each, and the specimens were then incubated in 70, 90 and 100% ice-cold ethanol and dried. Fifty milliliters of 50% formamide/2 × SSC was poured into a humidified chamber and preheated at 37 °C. The FITC-labeled circWAC probe and PE-labeled miR-142 probe (Tingzhou, Shanghai, China) were detected and observed by confocal microscopy. The probe sequences are shown in Supplementary Table 2.

### Nucleocytoplasmic fractionation

MDA-MB-231 cells were seeded in a 10 cm × 10 cm culture dish, and the cells were collected at 90% confluence. A PARIS kit was used to separate the nuclear and cytoplasmic components, and the cells were trypsinized after washing with PBS. The cells were transferred into a 2 mL EP tube and centrifuged at 500 r/min and 4 °C for 5 min, and the supernatant was then discarded. Then, 500 μL of fractionation buffer was added and gently mixed by pipetting, and the samples were placed on ice for 5 min and then centrifuged again under the same conditions. The supernatant was the cytoplasmic fraction, and the precipitate was the nuclear fraction. RNA extraction and quantitative analysis were performed on the nuclear and cytoplasmic fractions. U6 was used as the internal reference for the nuclear fraction, and GAPDH was used for the cytoplasmic fraction.

### RNA immunoprecipitation (RIP)

RNA enrichment was evaluated by qRT-PCR using a Magna RIP RNA binding protein immunoprecipitation kit (Millipore, Billerica, MA, USA) according to the manufacturer’s instructions with an anti-Ago2 antibody or IgG as the control.

### Immunohistochemical staining (IHC)

Paraffin tissue blocks were cut into 4 μm slices and mounted on a glass slide. The slices were dewaxed in xylene and hydrated through a graded series of ethanols. Then, the slices were immersed in a citric acid antigen retrieval solution and incubated in a microwave oven for 15 min for antigen retrieval. The samples were incubated with a 3% (v/v) H_2_O_2_ at 37 °C for 15 min to inhibit endogenous peroxidase activity, after which they were blocked using 10% goat serum and incubated at room temperature for 30 min. Then, the slices were incubated with a rabbit anti-human WWP1 monoclonal antibody overnight at 4 °C. The negative control slice was treated with phosphate-buffered saline (PBS) for 2 h under the same conditions. Then, the samples were incubated with the secondary antibody for 1 h prior to color development (ZSGB-Bio) and counterstaining with hematoxylin for 2 min. The results were assessed by the semiquantitative integration method. Five high-power fields were randomly selected in the area highly populated with cells, and 100 cells were counted. The percentage of positively stained cells was scored as follows: < 5% positively stained cells, 0 points; 5–25%, 1 point; 26–50%, 2 points; 51–75%, 3 points; and more than 75%, 4 points. The staining intensity score was assigned as follows: no staining, 0 points; light brownish-yellow staining, 1 point; brownish-yellow staining, 2 points; and brown staining, 3 points. The immunohistochemical score was calculated as the product of the positive percentage score and the staining intensity score.

### Animal experiments

Lentiviral vectors were used to generate MDA-MB-231/sh-NC cells and MDA-MB-231/sh-circWAC cells, which had downregulated circWAC expression. sh-NC (0.2 mL, 1 × 10^7^) and sh-circWAC (1 × 10^7^) cells were injected into the backs of BALB/c nude mice to generate 10 mice bearing implanted tumors. The size of the implanted tumors was observed every 3 d, and the volume of the tumors was calculated as (V) = (LxW^2^)/2. On d 21, the mice were randomly divided into 4 groups: sh-NC + control, sh-circWAC + control, MDA-MB-231/sh-NC + PTX, and sh-circWAC + PTX. Mice in the PTX group received intravenous injection of PTX (20 mg/kg) every 3 d for 5 cycles. After 18 d of treatment, all nude mice were sacrificed, and the tumor tissues were removed for evaluation of indexes.

### Statistical methods

Statistical analysis was performed using GraphPad Prism 6.0. The experimental data are expressed as the mean ± standard error of the mean (mean ± SEM) values. The *t* test was used to evaluate differences between two groups, and one-way analysis of variance (ANOVA) was used to evaluate differences among multiple groups. *p* < 0.05 was considered to be statistically significant, and each experiment was repeated at least 3 times.

## Results

### Differential expression of circRNAs in TNBC

**Fig. 1:**
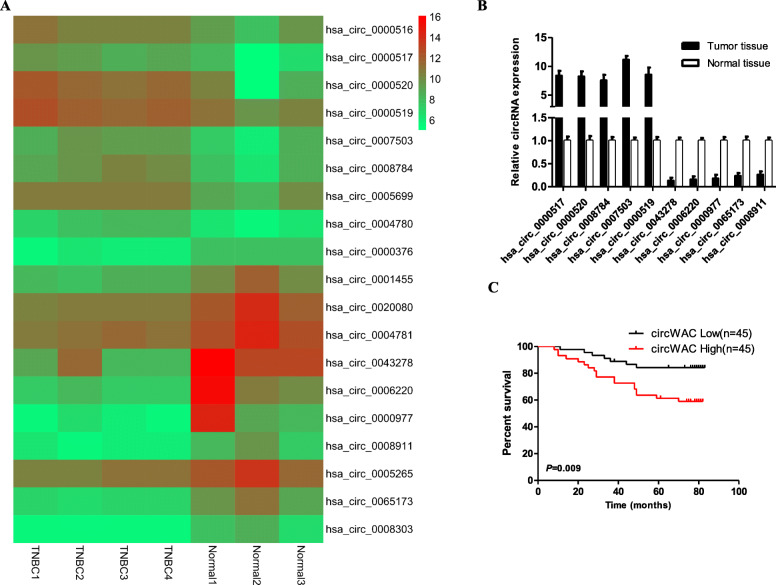
The expression and prognostic significance of circWAC in TNBC. **a** The heat map shows the 19 most upregulated and downregulated circRNAs in TNBC tissues compared with normal breast tissues in the GSE101124 dataset. **b** The top five upregulated circRNAs and top five downregulated circRNAs in TNBC tissues were verified by qRT-PCR. **c** Kaplan-Meier survival curves indicating the correlation between circWAC and overall survival in TNBC. ^*^P < 0.05, ^**^P < 0.01, ^***^P < 0.001

To study the differentially expressed circRNAs in TNBC tumor tissues and normal breast tissues, the GSE101124 dataset from the GEO database was used [10]. GSE101124 contains high-throughput array data of circRNAs in 4 TNBC tumor tissues and 3 normal breast tissues (Agilent-069978 Arraystar Human CircRNA microarray V1). With a fold change> 3.0 and a t test *p* < 0.05 as the threshold criteria, 8 upregulated circRNAs (hsa_circ_0000516, hsa_circ_0000517, hsa_circ_0000520, hsa_circ_0000519, hsa_circ_0007503, hsa_circ_0008784, hsa_circ_0005699 and hsa_circ_0004780) and 11 downregulated circRNAs (hsa_circ_0000376, hsa_circ_0001455, hsa_circ_0020080, hsa_circ_0004781, hsa_circ_0043278, hsa_circ_0006220, hsa_circ_0000977, hsa_circ_0008911, hsa_circ_0005265, hsa_circ_0065173 and hsa_circ_0008303) were identified (Fig. 1a). From the above circRNAs, we selected the 5 most upregulated circRNAs (hsa_circ_0000517, hsa_circ_0000520, hsa_circ_0008784, hsa_circ_0007503, hsa_circ_0000519) and the 5 most downregulated circRNAs (hsa_circ_0043278, hsa_circ_0006220, hsa_circ_0000977, hsa_circ_0065173 and hsa_circ_0008911) for further research. Thirty samples of TNBC tumor tissues and adjacent tissues were randomly selected from the biological sample bank of the First Affiliated Hospital of Soochow University for qRT-PCR analysis. The results showed that the expression of hsa_circ_0007503 in TNBC tumor tissues was significantly higher than that in adjacent tissues. This circRNA was upregulated 11.33-fold and had the highest uniformity and the most significantly different expression (Fig. 1b). As a result, hsa_circ_0007503 in TNBC was initially selected as the research object, and its clinical significance was evaluated. Hsa_circ_0007503 originates from the WAC gene and is hereafter referred to as circWAC.

To study the clinical significance of the expression of circWAC in TNBC, qRT-PCR was used to detect the correlation between the expression level of circWAC in 90 samples of TNBC and the clinicopathological characteristics of the corresponding patients. The cutoff criterion for high and low expression of the circRNA was the median value. The results of statistical analysis showed that circWAC was not significantly related to patient age, tumor differentiation, tumor size, or lymph node metastasis. However, the expression level of circWAC was significantly related to the prognosis of the TNBC patients. The higher the expression of circWAC, the worse was the overall survival (OS) of patients (Fig. 1c).

### Features of circWAC

**Fig. 2:**
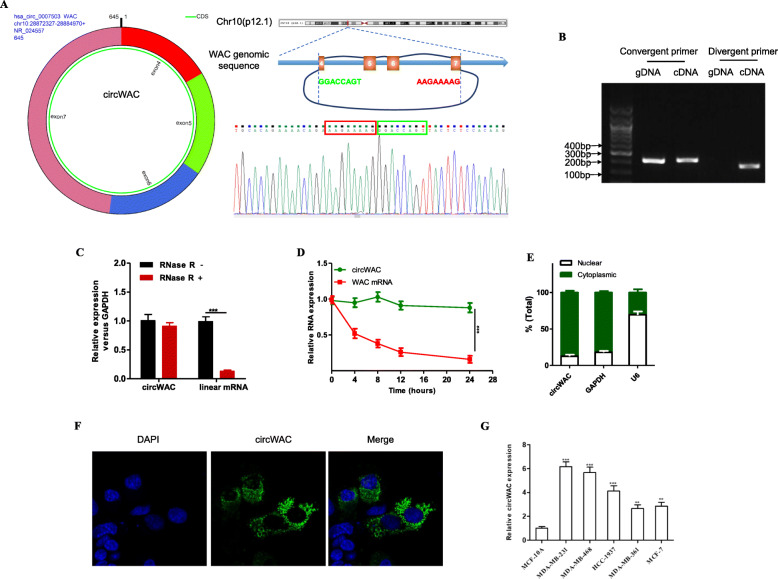
Characterization of circWAC as a circRNA in TNBC. **a** Information about the exonic structure of circWAC is illustrated as indicated. The specific primers used for validation of circWAC by Sanger sequencing. **b** qRT-PCR products generated with divergent primers showing circularization of circWAC. cDNA, complementary DNA. gDNA, genomic DNA. **c** qRT–PCR analysis of the expression of circWAC and WAC mRNA in MDA-MB-231 cells after treatment with RNase R. **d** qRT–PCR analysis of the expression of circWAC and WAC mRNA in MDA-MB-231 cells after treatment with actinomycin D at the indicated time points. **e** Nucleocytoplasmic fractionation experiment showing that circWAC was mainly distributed in the cytoplasm. **f** FISH analysis of circWAC. Nuclei were stained with DAPI. **g** The expression level of circWAC in a normal breast cell line (MCF-10A) and breast cancer cell lines was determined by qRT-PCR. The data are presented as the means±S.D. of at least three independent experiments. ^*^P < 0.05, ^**^P < 0.01, ^***^P < 0.001

Databases such as circBase (http://www.circbase.org/) and the UCSC Genome Browser (http://genome.ucsc.edu/) showed that circWAC is formed by the circularization of exons 4–7 of the WAC gene, that the spliced length is 645 bp and that it is an exon-derived circRNA. The results of Sanger sequencing confirmed head-to-tail splicing in the qRT-PCR product of circWAC (Fig. 2a). To prove that circWAC is circular rather than linear, we designed divergent and convergent primers for circWAC and the corresponding linear mRNA, respectively. Then, cDNA and gDNA were assayed by nucleic acid electrophoresis, and the results showed that circWAC is circular rather than linear (Fig. 2b). In addition, to confirm the stability of circWAC, we used RNase R treatment. Resistance to RNase R confirmed that circWAC has a circular structure (Fig. 2c). The results of actinomycin D treatment showed that the half-life of circWAC exceeded 24 h, whereas that of the associated linear transcript was approximately 4 h (Fig. 2d). To determine the intracellular distribution of circWAC, we performed a nucleocytoplasmic fractionation experiment and found that circWAC is mainly distributed in the cytoplasm (Fig. 2e). Fluorescence in situ hybridization (FISH) showed the same result (Fig. 2f). The expression of circWAC was evaluated in a normal breast cell line (MCF-10A) and breast cancer cell lines (MDA-MB-231, MDA-MB-468, HCC-1937, MDA-MB-361, MCF-7). The results showed that circWAC was highly expressed in breast cancer cell lines and was most highly expressed in TNBC cell lines (MDA-MB-231, MDA-MB-468, HCC-1937) (Fig. 2g). The two TNBC cell lines with the highest expression levels of circWAC (MDA-MB-231 and MDA-MB-468) were selected for subsequent cell experiments.

### Function of circWAC in TNBC

**Fig. 3:**
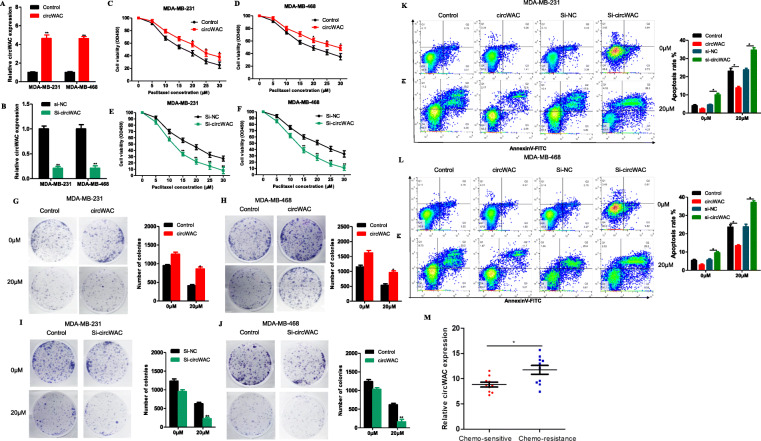
CircWAC decrease chemosensitivity to PTX in TNBC. **a**, **b** The efficiency of circWAC overexpression and silencing in MDA-MB-231 and MDA-MB-468 cells was verified by qRT-PCR. **c**-**f** Cells were treated with PTX (concentrations of 0, 5, 10, 15, 20, 25 and 30 μmol/L) for 24 h, and cell viability was evaluated by a CCK-8 assay. **g**-**j** Cells were treated with paclitaxel (0 and 20 μM) for 24 h, and cell viability was evaluated by a colony formation assay. **k**, **l** Cells were treated with paclitaxel (0 and 20 μM) for 24 h, and the apoptosis rate was analyzed via flow cytometry. The data are presented as the means±S.D. of at least three independent experiments. **m** The expression of circWAC was significantly increased in the chemotherapy resistance patients. ^*^P < 0.05, ^**^P < 0.01, ^***^P < 0.001

To study the function of circWAC in TNBC, the expression of circWAC was downregulated in the MDA-MB-231 cell line, and the changes in cell functions were observed. The experimental data showed that circWAC had no obvious relationship with cell metastasis and invasion (Supplementary figure 1) but was closely related to the chemosensitivity of cells. Then, the role of circWAC in chemosensitivity was investigated in MDA-MB-231 and MDA-MB-468 cells. MDA-MB-231 and MDA-MB-468 cells were transfected with a circWAC overexpression plasmid or siRNA. The expression levels of circRNA in cells after transfection are shown in Fig. 3a, b. The transfected cells were treated with different concentrations of PTX (0, 5, 10, 15, 20, 25, 30 μmol/L) for 24 h, and a CCK-8 assay was used to evaluate cell survival. Upregulation of circWAC decreased the sensitivity of cells to PTX (Fig. 3c, d), while downregulation of circWAC increased the sensitivity of cells to PTX (Fig. 3e, f). The IC50 values of PTX in MDA-MB-231 and MDA-MB-468 cells were 16.56 μmol/L and 20.31 μmol/L, respectively. After overexpression of circWAC, the IC50 values​ of PTX in MDA-MB-231 and MDA-MB-468 cells increased to 23.43 μmol/L and 29.51 μmol/L, respectively. After downregulation of circWAC, the IC50 values ​​of PTX in MDA-MB-231 and MDA-MB-468 decreased to 11.42 μmol/L and 12.43 μmol/L, respectively. Subsequent colony formation assays and apoptosis assays also confirmed that upregulation of circWAC reduced the sensitivity of cells to PTX, while downregulation of circWAC increased the sensitivity of cells to PTX (Fig. 3g-l). We also confirmed that circWAC could indeed increase the resistance of tumor cells to cisplatin (Supplementary Figure 2). In addition, we used 20 specimens from patients with metastatic TNBC in our specimen library. Ten patients were evaluated as CR or PR with target lesion reduction greater than 50% after 4 cycles of PTX-based chemotherapy, and they were deemed sensitive to PTX chemotherapy. The other 10 patients with tumor progression or reduction of less than 30% after 4 cycles of PTX-based chemotherapy were regarded as chemotherapy resistance. We found that the expression of circWAC was significantly increased in the chemo-resistance group (Fig. 3m).

### CircWAC regulates PTX resistance by targeting miR-142

**Fig. 4:**
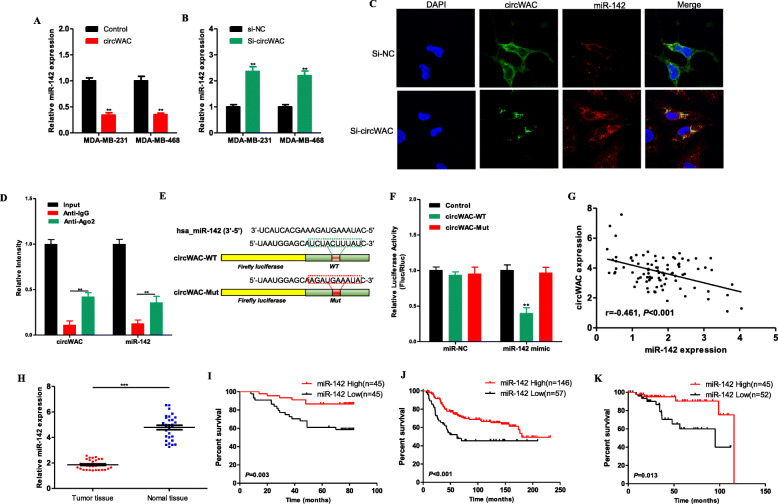
CircWAC acts as a sponge of miR-142 in TNBC. **a** The expression level of miR-142 in MDA-MB-231 and MDA-MB-468 cells transfected with circWAC or control. **b** The expression level of miR-142 in MDA-MB-231 and MDA-MB-468 cells transfected with si-circWAC or si-NC. **c** FISH showed that circWAC and miR-142 were colocalized in the cytoplasm of MDA-MB-231 cells. **d** The coprecipitated miR-142 was subjected to qRT-PCR to assess circWAC binding by RIP experiments in MDA-MB-231 cells. **e** MiR-142 and wild-type/mutant circWAC binding site sequences. **f** Luciferase activity in MDA-MB-231 cells cotransfected with a luciferase reporter vector containing the wild-type or mutant circWAC sequence and the miR-142 mimic or control. **g** The expression of miR-142 was negatively correlated with circWAC in 90 TNBC patients. **h** The expression level of miR-142 in TNBC tissues and adjacent nontumor tissues was measured by qRT-PCR. Kaplan-Meier survival analysis of patients with TNBC stratified by the level of miR-142 were used to evaluate OS based on our dataset (*N* = 90) (I), a METABRIC dataset (*N* = 203) (J) and a TCGA dataset (*N* = 97) (K)

The competing endogenous RNA (ceRNA) mechanism, which mainly regulates gene expression at the posttranscriptional level, is currently the most widely reported circRNA regulatory mechanism. The online analysis software associated with the Circular RNA Interactome (https://circinteractome.nia.nih.gov/) and circBANK (http://www.circbank.cn/) databases was used for bioinformatic analysis to screen miRNAs according to their RNA binding scores. It was found that miR-593, miR-383, miR-498, miR-142-5p (hereafter referred to as miR-142) and miR-1183 are miRNAs potentially sponged by circWAC. Subsequently, the expression level of circWAC in MDA-MB-231 and MDA-MB-468 cells was upregulated or downregulated, and among the above identified miRNAs, only the expression level of miR-142 changed (Fig. 4a, b), suggesting that miR-142 is a miRNA potentially sponged by circWAC. To observe the colocalization of circWAC and miR-142 in cells, FISH was performed. The results showed that circWAC and miR-142 colocalized in MDA-MB-231 cells mainly in the cytoplasm (Fig. 4c). RNA immunoprecipitation was performed to observe whether circWAC can bind to miR-142. The RIP results showed higher levels of circWAC and miR-142 in the Ago2 antibody group than in the IgG control group (Fig. 4d), proving that circWAC can bind to miR-142. Then, plasmids containing the wild-type sequence (circWAC-WT) and the mutant binding site sequence (circWAC-MUT) were constructed (Fig. 4e) and cotransfected with the miR-142 mimic or miR-NC into MDA-MB-231 cells for a dual luciferase reporter assay. The results showed that overexpression of miR-142 significantly reduced the luciferase activity of the circWAC-WT vector but did not reduce the luciferase activity of the empty vector or the circWAC-MUT vector (Fig. 4f), confirming that circWAC can directly interact with miR-142. In addition, we performed a correlation analysis between circWAC and miR-142 based on 90 TNBC tissues. The expression of miR-142 was negatively correlated with circWAC in TNBC (Fig. 4g).

MiR-142 has been confirmed to play a role as a tumor suppressor in a variety of cancers [11, 12]. The expression level of miR-142 was evaluated by qRT-PCR in 30 paired TNBC tumor tissues and adjacent tissue samples. The expression level of miR-142 in tumor tissues was significantly lower than that in adjacent nontumor tissues (Fig. 4h). Then, the expression level of miR-142 in 90 samples of TNBC was analyzed by qRT-PCR, and the survival outcomes of the corresponding patients were analyzed. The median expression level was used as the cutoff value, and it was found that the OS of TNBC patients with high miR-142 expression was significantly better than that of patients with low miR-142 expression (Fig. 4i). Subsequently, the data of TNBC patients in the METABRIC and TCGA databases were used for verification (the relevant data were downloaded from Kaplan-Meier Plotter, http://kmplot.com/analysis/), and the same results were obtained (Fig. 4j, k).

**Fig. 5:**
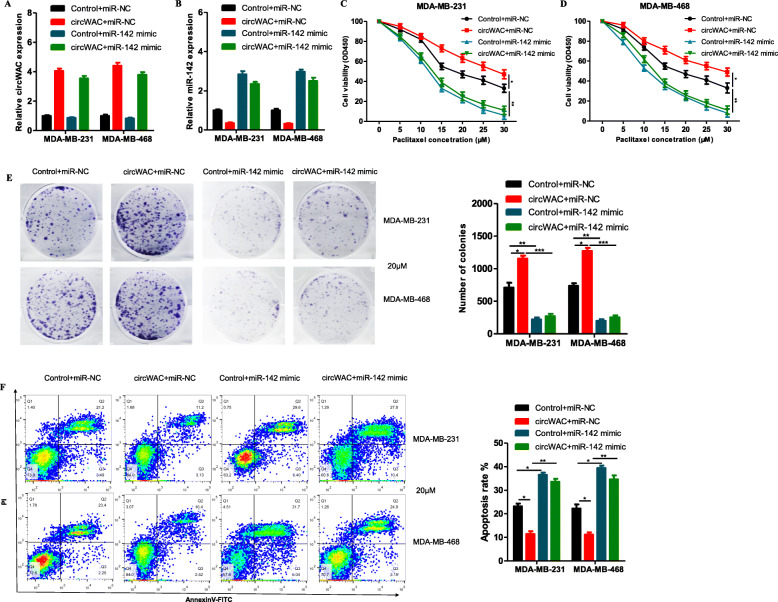
CircWAC regulates PTX resistance by targeting miR-142. **a**, **b** The expression of circWAC and miR-142 in MDA-MB-231 and MDA-MB-468 cells transfected with control + miR-NC, circWAC + miR-NC, control+miR-142 mimic, or circWAC+miR-142 mimic was verified by qRT-PCR. **c**, **d** MDA-MB-231 and MDA-MB-468 cells transfected with control + miR-NC, circWAC + miR-NC, control+miR-142 mimic, or circWAC+miR-142 mimic were treated with PTX (concentrations of 0, 5, 10, 15, 20, 25 and 30 μmol/L) for 24 h, and cell viability was evaluated by a CCK-8 assay. **e** MDA-MB-231 and MDA-MB-468 cells transfected with control + miR-NC, circWAC + miR-NC, control+miR-142 mimic, or circWAC+miR-142 mimic were treated with 20 μM paclitaxel for 24 h, and cell viability was evaluated by a colony formation assay. **f** MDA-MB-231 and MDA-MB-468 cells transfected with control + miR-NC, circWAC + miR-NC, control+miR-142 mimic, or circWAC+miR-142 mimic were treated with 20 μM paclitaxel for 24 h, and the apoptosis rates were analyzed via flow cytometry. The data are presented as the means±S.D. of at least three independent experiments. ^*^P < 0.05, ^**^P < 0.01, ^***^P < 0.001

To determine whether circWAC regulates breast cancer chemosensitivity through miR-142, a gain-of-function assay was conducted. MDA-MB-231 and MDA-MB-468 cells were transfected to generate the following 4 groups: (1) control + miR-NC, (2) circWAC + miR-NC, (3) control + miR-142 mimic, and (4) circWAC + miR-142 mimic. qRT-PCR was then used to verify the expression of circWAC and miR-142 after transfection (Fig. 5a, b). The transfected cells were incubated with different concentrations of PTX (0, 5, 10, 15, 20, 25, 30 μmol/L) for 24 h, and a CCK-8 assay was used to evaluate cell survival. The results showed that overexpression of circWAC reduced the PTX sensitivity of cells compared with that in the control group (Fig. 5c, d). In contrast, overexpression of miR-142 increased the sensitivity of the cells to PTX (Fig. 5c, d). However, in cells overexpressing miR-142, upregulation of circWAC did not reverse the chemosensitivity induced by miR-142 (Fig. 5c, d). Subsequent colony formation assays and apoptosis assays conducted with a PTX concentration of 20 μmol/L confirmed these results (Fig. 5e, f). Therefore, it is believed that miR-142 can block the induction of chemotherapeutic resistance by circWAC, indicating that the role of circWAC in PTX resistance in TNBC cells must depend on miR-142.

### WWP1 was the direct target gene of miR-142

**Fig. 6:**
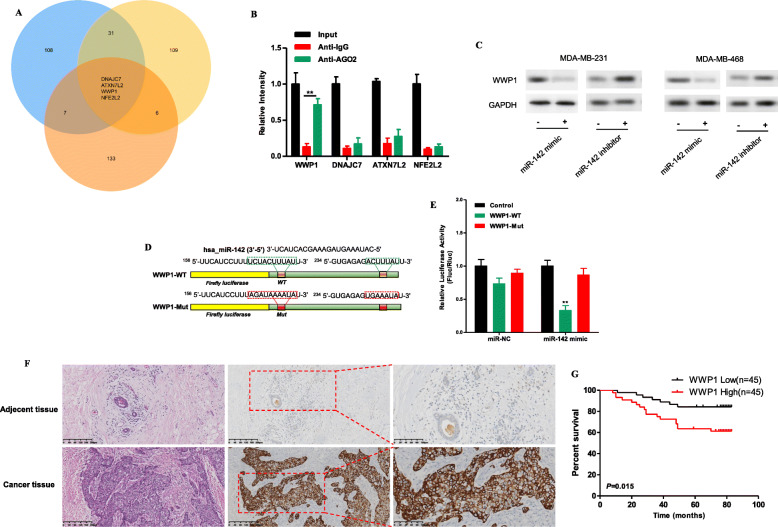
WWP1 is the direct target gene of miR-142. **a** TargetScan, miRDB and miRNAMAP were used to predict the potential target genes of miR-142. **b** RIP experiments were performed in MDA-MB-231 cells, and the coprecipitated miRNA was subjected to qRT-PCR to evaluate mRNA binding. **c** The protein expression of WWP1 in MDA-MB-231 and MDA-MB-468 cells transfected with the miR-142 mimic or miR-142 inhibitor was measured by Western blotting. **d** MiR-142 and wild-type/mutant WWP1 binding site sequences. **e** Luciferase activity in MDA-MB-231 cells cotransfected with a luciferase reporter vector containing the wild-type or mutant WWP1 sequence and the miR-142 mimic or control. **f** H&E staining was performed to evaluate tissue morphology, and IHC was performed to evaluate WWP1 expression in TNBC tissue and adjacent tissue. **g** Kaplan-Meier survival curves indicating the correlation between WWP1 expression and overall survival in TNBC. ^*^P < 0.05, ^**^P < 0.01, ^***^P < 0.001

TargetScan (http://www.targetscan.org/), miRDB (http://www.mirdb.org/) and miRNAMAP (http://mirnamap.mbc.nctu.edu.tw/) were used to predict the potential target genes of miR-142. The top 150 target genes predicted by the three databases were analyzed, and the 4 most promising target genes of miR-142 predicted by all three databases were identified: WWP1, DNAJC7, ATXN7L2 and NFE2L2 (Fig. 6a). These genes were evaluated with a RIP assay in MDA-MB-231 cells to determine whether their mRNA can bind to miR-142. The results showed that compared with that in the IgG control group, the mRNA level of WWP1 in the Ago2 antibody group was significantly increased (Fig. 6b). Subsequently, the miR-142 mimic or miR-142 inhibitor was transfected into MDA-MB-231 and MDA-MB-468 cells, and Western blotting was used to detect the changes in WWP1 protein expression levels. It was found that overexpression of miR-142 decreased the protein expression level of WWP1, while downregulation of miR-142 increased the protein expression level of WWP1 (Fig. 6c). Then, plasmids containing the wild-type sequence (WWP1-WT) or the mutant binding site sequence (WWP1-MUT) were constructed (Fig. 6d) and cotransfected with the miR-142 mimic or miR-NC into MDA-MB-231 cells for a dual luciferase reporter assay. The results showed that overexpression of miR-142 significantly reduced the luciferase activity of the vector containing WWP1-WT but did not reduce the luciferase activity of the empty vector or the vector containing WWP1-MUT (Fig. 6e), confirming that WWP1 is the direct target gene of miR-142.

WWP1 has been confirmed to act as an oncogene in a variety of cancers [13–18], including breast cancer [15–18]. High expression of WWP1 regulates PTEN polyubiquitination, thereby promoting activation of the PI3K/AKT signaling pathway [14]. The protein expression level of WWP1 in 90 TNBC samples was examined by IHC, and it was found that WWP1 expression in TNBC tissues was higher than that in adjacent normal tissues (Fig. 6f). Survival analysis was subsequently performed, and the median expression level was used as the cutoff value. The results indicated that the OS of TNBC patients with high WWP1 expression was significantly poorer than that of patients with low WWP1 expression (Fig. 6g).

**Fig. 7:**
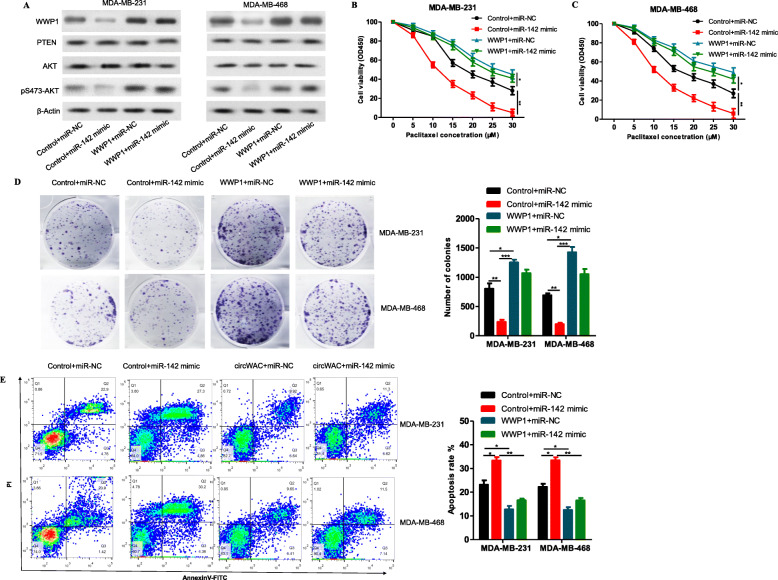
MiR-142 increases PTX sensitivity and inhibits the PI3K/AKT signaling pathway by targeting WWP1. **a** The protein levels of WWP1, PTEN, AKT and pS473-AKT in MDA-MB-231 and MDA-MB-468 cells transfected with control + miR-NC, control + miR-142 mimic, WWP1 + miR-NC, or WWP1 + miR-142 mimic were measured by Western blotting. **b**, **c** MDA-MB-231 and MDA-MB-468 cells transfected with control + miR-NC, control + miR-142 mimic, WWP1 + miR-NC, or WWP1 + miR-142 mimic were treated with PTX (concentrations of 0, 5, 10, 15, 20, 25 and 30 μmol/L) for 24 h, and cell viability was evaluated by a CCK-8 assay. **d** MDA-MB-231 and MDA-MB-468 cells transfected with control + miR-NC, control + miR-142 mimic, WWP1 + miR-NC, or WWP1 + miR-142 mimic were treated with 20 μM paclitaxel for 24 h, and cell viability was evaluated by a colony formation assay. **e** MDA-MB-231 and MDA-MB-468 cells transfected with control + miR-NC, control + miR-142 mimic, WWP1 + miR-NC, or WWP1 + miR-142 mimic were treated with 20 μM paclitaxel for 24 h, and the apoptosis rates were analyzed via flow cytometry. The data are presented as the means±S.D. of at least three independent experiments. ^*^P < 0.05, ^**^P < 0.01, ^***^P < 0.001

**Fig. 8:**
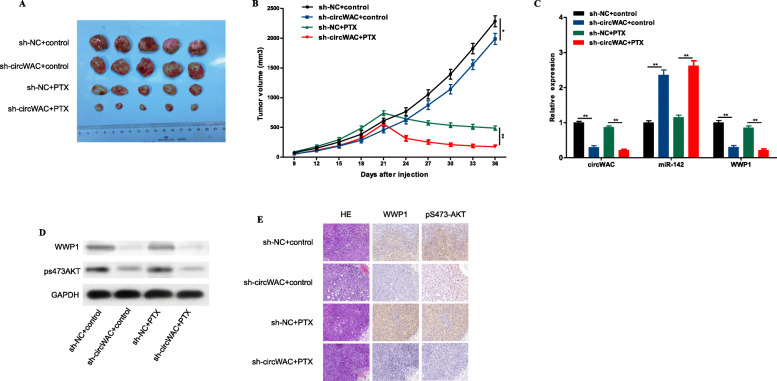
A summary map to illustrate the interaction relation among these molecules and signal pathways to drug resistance in TNBC

To determine whether miR-142 regulates breast cancer chemosensitivity through WWP1, a gain-of-function assay was conducted. MDA-MB-231 and MDA-MB-468 cells were transfected to generate the following 4 groups: (1) control + miR-NC, (2) control + miR-142 mimic, (3) WWP1 + miR-NC, and (4) WWP1 + miR-142 mimic. Western blot analysis was used to verify the levels of WWP1, PTEN, AKT and pS473-AKT after transfection. The results showed that overexpression of WWP1 induced the phosphorylation of AKT at S473 (Fig. 7a), indicating that WWP1 overexpression can activate the PI3K/AKT signaling pathway, while overexpression of miR-142 inhibited the expression of WWP1 and the phosphorylation of AKT at S473 (Fig. 7a), indicating that miR-142 overexpression can inhibit the PI3K/AKT signaling pathway. The transfected cells were incubated with different concentrations of PTX (0, 5, 10, 15, 20, 25, 30 μmol/L) for 24 h, and a CCK-8 assay was used to evaluate cell survival. The results showed that overexpression of WWP1 could reduce the PTX sensitivity compared with that in the control group. In WWP1-overexpressing cells, upregulation of miR-142 could not reverse the chemotherapeutic resistance induced by WWP1 (Fig. 7b, c). Subsequent colony formation assays and apoptosis assays conducted with a PTX concentration of 20 μmol/L confirmed these results (Fig. 7d, e). Therefore, it is believed that WWP1 can block the induction of chemosensitivity by miR-142, indicating that the role of miR-142 in PTX resistance in TNBC cells must depend on WWP1. In summary, circWAC is an oncogene acting as a ceRNA to indirectly upregulate the expression of the target gene of miR-142, WWP1, by competitively binding miR-142 and activating the PI3K/AKT pathway (Fig. 8).

### Inhibition of circWAC increases sensitivity to PTX in vivo

**Fig. 9:**
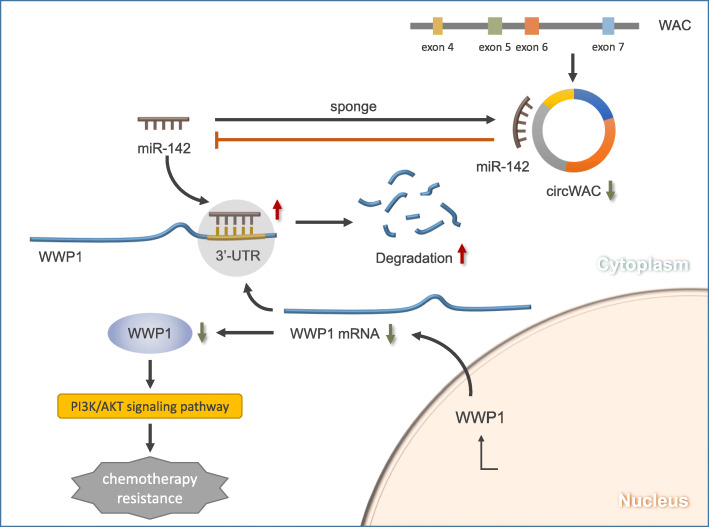
Inhibition of circWAC can increase chemosensitivity to PTX in vivo. **a** Image of representative resected tumors from four groups of xenografted nude mice on the 36th day. **b** Tumor volumes were monitored during the 36-d time course. **c** The expression levels of circWAC, miR-142 and WWP1 in tumor tissues were determined by qRT-PCR. **d** The levels of WWP1 and pS473-AKT in tumor tissues were detected by Western blotting. **e** H&E staining was performed to evaluate tissue morphology, and IHC was performed to visualize WWP1 and pS473-AKT. ^*^P < 0.05, ^**^P < 0.01, ^***^P < 0.001

To verify whether inhibiting circWAC in TNBC cells can increase their sensitivity to PTX, the following animal experiment was conducted. Mice were divided into the following four groups (see the Methods section for details): sh-NC + control, sh-circWAC + control, sh-NC + PTX, and sh-circWAC + PTX. The tumors in sh-circWAC + PTX group mice were significantly smaller than those in sh-NC + PTX group mice, suggesting that inhibition of circWAC significantly increased chemosensitivity to PTX in the mice (Fig. 9a, b). Then, the expression of circWAC, miR-142 and WWP1 was evaluated in tumor tissues by qRT-PCR. It was found that circWAC and WWP1 were downregulated while miR-142 was highly expressed in the sh-circWAC and sh-circWAC + PTX groups, indirectly proving that circWAC competitively binds to miR-142 to upregulate the expression of WWP1 (Fig. 9c). Then, IHC and Western blotting were used to detect the levels of WWP1 and pS473-AKT in tumor tissues, and it was found that WWP1 and pS473-AKT were expressed at low levels in the sh-circWAC and sh-circWAC +PTX groups (Fig. 9d, e), proving that decreased expression of WWP1 causes inactivation of the PI3K/AKT signaling pathway and leads to an increase in the sensitivity of breast cancer cells to chemotherapy.

## Discussion

In the treatment of TNBC, PTX-based regimens have proven to be an important chemotherapeutic approach. Mechanistically, PTX treatment acts by inducing mitotic arrest by stabilizing spindle-shaped microtubules, which are responsible for the separation of replicated chromosomes into the daughter cell. However, the application of PTX-based chemotherapy in TNBC is limited by the emergence of PTX resistance. As noncoding molecules, circRNAs have been shown to play important roles in the occurrence and development of tumors [19]. Previous reports have identified a number of abnormally expressed circRNAs in TNBC that are upregulated in TNBC tissues and function as oncogenes. For example, circGFRA1 can promote tumor proliferation and inhibit apoptosis by binding to miR-34a and upregulating the expression of GFRA1 in TNBC [20]. CircEPSTI1 can increase BCL11A expression in TNBC by sponging miR-4753 and miR-6809, thus promoting cell proliferation and inhibiting apoptosis [21]. Zeng et al. found that circANKS1B is significantly upregulated in TNBC and that upregulation of circANKS1B expression is closely related to lymph node metastasis [22]. In vitro experiments showed that circANKS1B upregulates the expression of USF1 by sponging miR-148a-3p and miR-152-3p, which leads to upregulation of TGF-β1/Smad signaling and promotes EMT [22]. In addition, it was found that circAGFG1, circKIF4A, circPLK1 and circRAD18 can promote the malignant progression of TNBC [23–26]. Several studies have shown that circRNAs also play a role in suppressing TNBC. CircITCH is significantly downregulated in TNBC tissue, and downregulated circITCH expression is closely related to the poor prognosis of patients [27]. Overexpression of circITCH can upregulate ITCH1 by sponging miR-214 and miR-17, thereby inactivating Wnt/β-catenin signaling [27]. Xu et al. found that upregulating the expression of circTADA2A-E6 inhibits cell proliferation, migration and colony formation via regulation of the miR-203a-3p/SOCS3 signaling pathway [10]. CircFBXW7 inhibits the malignant progression of TNBC by upregulating miR-197-3p and encoding the tumor suppressor FBXW7-185aa [28]. These circRNAs involved in TNBC, which mostly affect the proliferation, invasion and metastasis of tumor cells through the ceRNA mechanism, are closely related to the clinicopathological factors of TNBC patients and have potential as prognostic indexes of TNBC. In this study, it was found that circWAC was highly expressed in TNBC and was associated with poor TNBC patient prognosis. Then, the results of experiments in cells and animal models verified that downregulation of circWAC increases the sensitivity of TNBC cells to PTX.

Next, the molecular mechanism of circWAC in TNBC chemotherapeutic resistance was explored and verified. First, candidate miRNAs with sequences complementary to circWAC were screened through bioinformatic approaches, and it was found that the expression of miR-142 was also negatively regulated by circWAC in TNBC cells. RNA FISH showed that circWAC and miR-142 colocalized in the cytoplasm of MDA-MB-231 cells. Furthermore, RIP and dual luciferase reporter assays proved a direct interaction between circWAC and miR-142. In the gain-of-function assay, miR-142 blocked the induction of chemotherapeutic resistance in TNBC cells by circWAC. In addition, the OS of TNBC patients with high expression of miR-142 was significantly better than that of patients with low expression of miR-142, and these results were verified in the METABRIC and TCGA databases. MiR-142 has been confirmed to play a role as a tumor suppressor in a variety of cancers [11, 12, 29, 30]. Low expression of miR-142 is closely related to the high recurrence and poor prognosis of gastric cancer [12]. Pancreatic cancer patients with high expression of miR-142 have longer OS times than those with low expression of this miRNA [29]. Tsang et al. found that miR-142 is expressed at significantly lower levels in HCC tissue than in non-HCC tissue and that high expression of miR-142 can inhibit the proliferation and migration of HCC cells [30]. Jia et al. found that miR-142 inhibitors can enhance the ability of lung adenocarcinoma cells to undergo EMT [11]. Finally, bioinformatic analyses and cell experiments were performed to identify target genes regulated by miR-142, and it was proven that both WWP1 expression and PI3K/AKT pathway activity were regulated by circWAC and miR-142. Gain-of-function assays confirmed that WWP1 can block the chemosensitivity of TNBC cells through miR-142. In addition, it was confirmed that WWP1 was highly expressed in TNBC and that the prognosis of patients with high WWP1 expression was poor. Previously, WWP1 was shown to be overexpressed or amplified in breast cancer, indicating that it acts as an oncogene [15–18]. Recently, WWP1 has been proven to act as a switch to reactivate PTEN. Downregulation of WWP1 leads to inactivation of the PI3K/AKT signaling pathway [13, 14].

## Conclusion

In summary, circWAC/miR-142/WWP1 can form a ceRNA network to regulate PI3K/AKT signaling activity in TNBC cells and affect their chemosensitivity. Based on this mechanism, it is believed that the circWAC/miR-142/WWP1 axis has great potential as a new biomarker and new therapeutic target for TNBC.

## Supplementary Information

**Additional file 1: Supplementary Table 1.** Primers for qRT-PCR. **Supplementary Table 2.** RNA probes for FISH.

**Additional file 2: Supplementary Figure 1.** A. Cell invasion ability of MDA-MB-231 cells transfected with Si-NC or Si-circWAC was evaluated by transwell invasion assays. B. Wound healing assays of MDA-MB-231 cells transfected with Si-NC or Si-circWAC were performed to evaluate cell migration ability. **Supplementary Figure 2.** A-D. Cells were treated with cisplatin (concentrations of 0, 2.5, 5, 7.5, 10 and 12.5 μmol/L) for 24 h, and cell viability was evaluated by a CCK-8 assay. E-H. Cells were treated with cisplatin (0 and 10 μM) for 24 h, and cell viability was evaluated by a colony formation assay. I-J. Cells were treated with cisplatin (0 and 10 μM) for 24 h, and the apoptosis rate was analyzed via flow cytometry. The data are presented as the means±S.D. of at least three independent experiments. *P < 0.05, **P < 0.01, ***P < 0.001.

## Data Availability

The circular RNA sequencing datasets were obtained from the Gene Expression Omnibus database (http://www.ncbi.nlm.nih.gov/geo/) under the series accession number GSE101124. MiR-142-related data from the METABRIC and TCGA databases were downloaded from Kaplan-Meier Plotter (http://kmplot.com/analysis/). All data and materials supporting the conclusions of this study have been included within the article and the supplemental data.

## Abbreviations

Ago: Argonaute 2
CCK-8: Cell Counting Kit-8
cDNA: Complementary DNA
ceRNAs: Competing endogenous RNAs
circRNAs: Circular RNAs
RNA FISH: RNA fluorescence in situ hybridization
gDNA: Genomic DNA
H&E: Hematoxylin and eosin
IHC: Immunohistochemical staining
OS: Overall survival
PTX: Paclitaxel
qRT-PCR: Quantitative real-time PCR
RIP: RNA immunoprecipitation
siRNAs: Short interfering RNAs
TNBC: Triple-negative breast cancer
WWP1: WW domain–containing ubiquitin E3 ligase 1
