# Supplementary material for: CircWAC induces chemotherapeutic resistance in triple-negative breast cancer by targeting miR-142, upregulating WWP1 and activating the PI3K/AKT pathway
Source: Mol Cancer. 2021 Mar 1;20:43. doi: 10.1186/s12943-021-01332-8 (PMC7919093; doi:10.1186/s12943-021-01332-8)
Supplement: Supplementary file 1 — Additional file 1: Supplementary Table 1. Primers for qRT-PCR. Supplementary Table 2. RNA probes for FISH. [file 12943_2021_1332_MOESM1_ESM.docx]

**Supplementary Table 1. Primers for qRT-PCR**

| **Gene name** | **Forward primer (5’-3’)** | **Reverse primer (5’-3’)** |
| --- | --- | --- |
| circWAC | ACTGCCTACACCCACATCTT | GTGGAGAGTAACTGGTCCCTT |
| hsa_circ_0000517 | CGCGAGGTCTGAGACTAGG | CAGGGAGAGCCCTGTTAGG |
| hsa_circ_0000520 | GGGGAGGGAAGCTCATCAG | ACATGGGAGTGGAGTGACAG |
| hsa_circ_0008784 | CATGGAGTGTGTGCCTTGTG | TGTTGGAGAGTTAGTGCGGT |
| hsa_circ_0000519 | GAGCTTCGGGGAGCTGAG | CAGACCTTCCCAAGGGACAT |
| hsa_circ_0043278 | AGCCATTCCATTTCACTACTTCA | CCACAGTCCATCACAGCTTC |
| hsa_circ_0006220 | AGCCATTCCATTTCACTACTTCA | CCACAGTCCATCACAGCTTC |
| hsa_circ_0000977 | TGGGCATCTATTACATTCCATTCTG | GTGACACTGTTTAAGGCGCA |
| hsa_circ_0065173 | TCCTTCAGAAGAAGAAAATGAGGC | TGCCAAGTGCAGTGAGAAAC |
| hsa_circ_0000977 | TGGGCATCTATTACATTCCATTCTG | TTCTCCGCAGCATCAGTTTG |
| WWP1 | TGCTTCACCAAGGTCTGATACT | GCTGTTCCGAACCAGTTCTTTT |
| DNAJC7 | AGCTATTATGGTAATCGAGCAGC | GGACAAAACTGTCATCCAACCTC |
| ATXN7L2 | GCAGTGTGGGGTAATAAATCCA | CAGCACGTCAAAGTCCTTGG |
| NFE2L2 | TCAGCGACGGAAAGAGTATGA | CCACTGGTTTCTGACTGGATGT |
| GAPDH | GGAGCGAGATCCCTCCAAAAT | GGCTGTTGTCATACTTCTCATGG |

The miRNA levels were assayed with Taqman probes and primer sets (Applied Biosystems, Foster City, CA, USA) in accordance with the manufacturer's instructions.

**Supplementary Table 2. RNA probes for FISH**

| **Gene name** | **probe sequence** |
| --- | --- |
| circWAC | CTTTCTTTTCCCTGGTCAATGAG |
| miR-142 | AGTAGTGCTTTCTACTTTATG |
