# Supplementary material for: CircWAC induces chemotherapeutic resistance in triple-negative breast cancer by targeting miR-142, upregulating WWP1 and activating the PI3K/AKT pathway
Source: Mol Cancer. 2021 Mar 1;20:43. doi: 10.1186/s12943-021-01332-8 (PMC7919093; doi:10.1186/s12943-021-01332-8)
Supplement: Supplementary file 2 — Additional file 2: Supplementary Figure 1. A. Cell invasion ability of MDA-MB-231 cells transfected with Si-NC or Si-circWAC was evaluated by transwell invasion assays. B. Wound healing assays of MDA-MB-231 cells transfected with Si-NC or Si-circWAC were performed to evaluate cell migration ability. Supplementary Figure 2. A-D. Cells were treated with cisplatin (concentrations of 0, 2.5, 5, 7.5, 10 and 12.5 μmol/L) for 24 h, and cell viability was evaluated by a CCK-8 assay. E-H. Cells were treated with cisplatin (0 and 10 μM) for 24 h, and cell viability was evaluated by a colony formation assay. I-J. Cells were treated with cisplatin (0 and 10 μM) for 24 h, and the apoptosis rate was analyzed via flow cytometry. The data are presented as the means±S.D. of at least three independent experiments. *P < 0.05, **P < 0.01, ***P < 0.001. [file 12943_2021_1332_MOESM2_ESM.pdf]

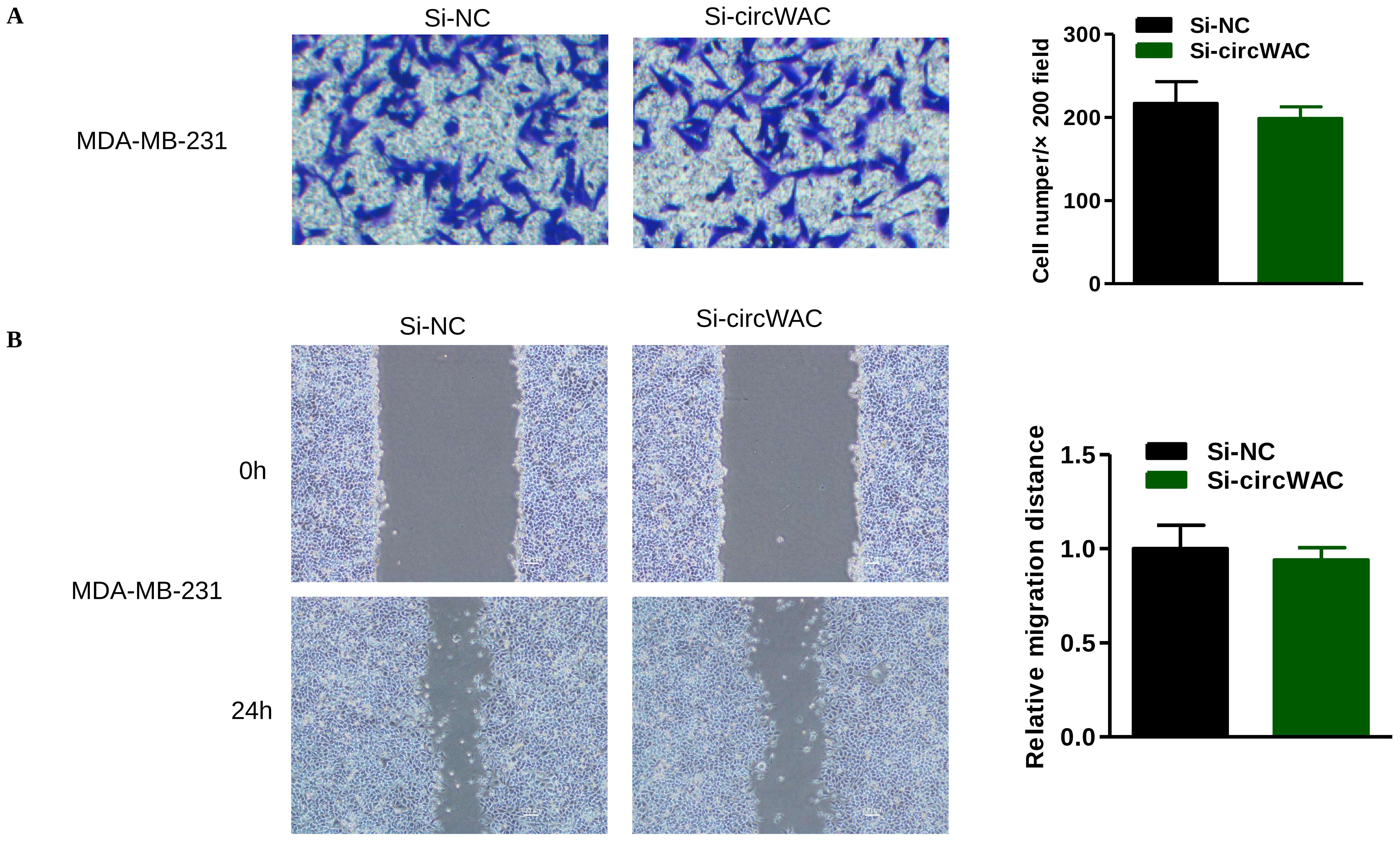

Supplementary figure 1. A. Cell invasion ability of MDA-MB-231 cells transfected with Si-NC or Si-circWAC was evaluated by transwell invasion assays. B. Wound healing assays of MDA-MB-231 cells transfected with Si-NC or Si-circWAC were performed to evaluate cell migration ability.

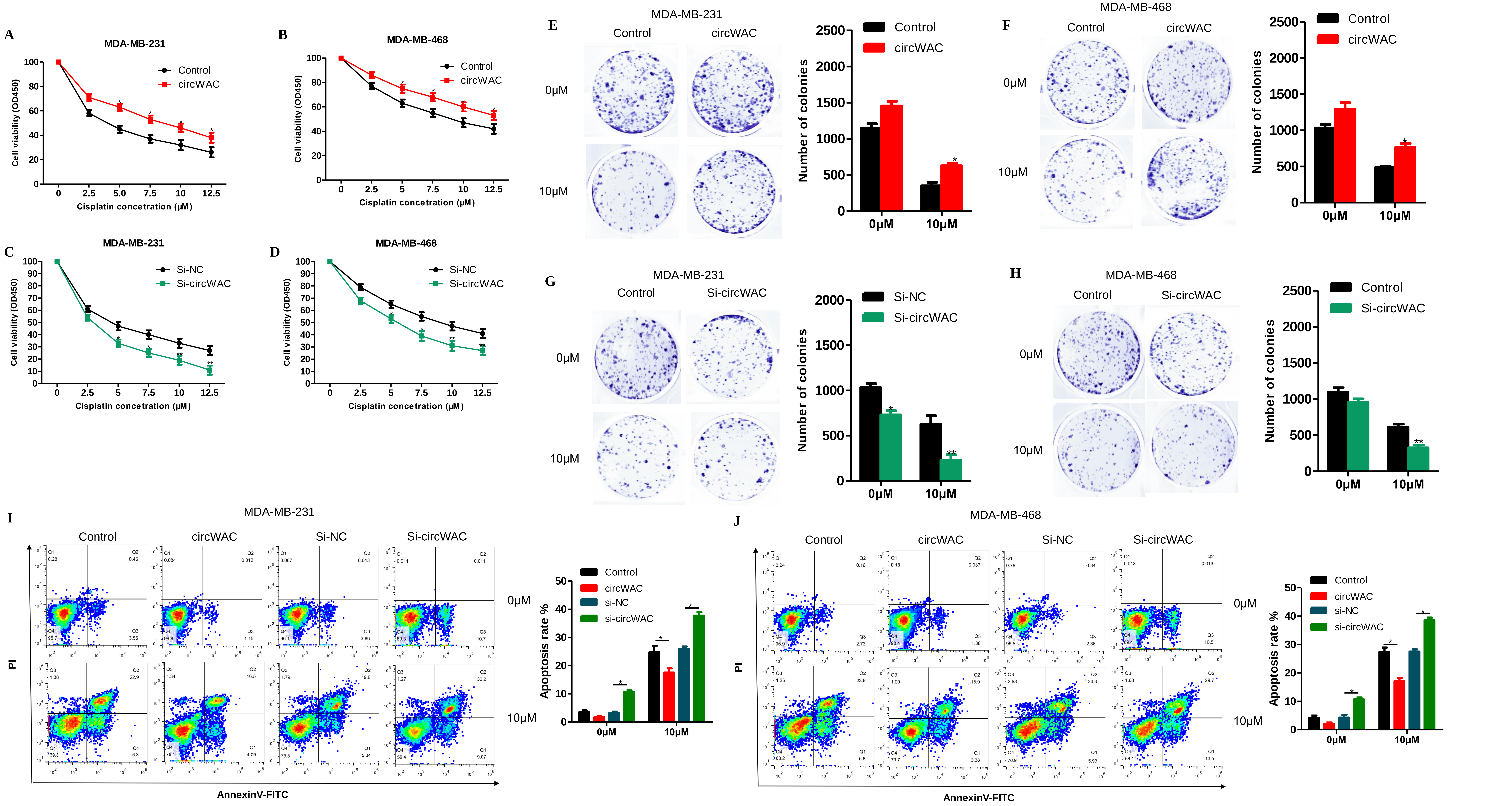

Supplementary figure 2. A-D. Cells were treated with cisplatin (concentrations of 0, 2.5, 5, 7.5, 10 and 12.5 μmol/L) for 24 h, and cell viability was evaluated by a CCK-8 assay. E-H. Cells were treated with cisplatin (0 and 10 μM) for 24 h, and cell viability was evaluated by a colony formation assay. I-J. Cells were treated with cisplatin (0 and 10 μM) for 24 h, and the apoptosis rate was analyzed via flow cytometry. The data are presented as the means±S.D. of at least three independent experiments. $^{*}P<0.05$, $^{**}P<0.01$, $^{***}P<0.001$.
